# A simple prognostic index based on admission vital signs data among patients with sepsis in a resource-limited setting

**DOI:** 10.1186/s13054-015-0826-8

**Published:** 2015-03-16

**Authors:** Stephen B Asiimwe, Amir Abdallah, Richard Ssekitoleko

**Affiliations:** Department of Medicine, Mbarara Regional Referral Hospital, P.O. Box 40, Mbarara, Uganda; Department of Medicine, Mbarara University of Science and Technology, P.O. Box 1410, Mbarara, Uganda; Department of Epidemiology and Biostatistics, University of California San Francisco, 550 16th Street, Second Floor, Mission Hall: Global Health and Clinical Sciences Building, San Francisco, CA 94158-2549 USA; Department of Medicine, College of Health Sciences, Makerere University, P.O. Box 7072, Kampala, Uganda

## Abstract

**Introduction:**

In sub-Saharan Africa, vital signs are a feasible option for monitoring critically ill patients. We assessed how admission vital signs data predict in-hospital mortality among patients with sepsis. In particular, we assessed whether vital signs data can be incorporated into a prognostic index with reduced segmentation in the values of included variables.

**Methods:**

Subjects were patients with sepsis hospitalized in Uganda, who participated in two cohort studies. Using restricted cubic splines of admission vital signs data, we predicted probability of in-hospital death in the development cohort and used this information to construct a simple prognostic index. We assessed the performance of the index in a validation cohort and compared its performance to that of the Modified Early Warning Score (MEWS).

**Results:**

We included 317 patients (167 in the development cohort and 150 in the validation cohort). Based on how vital signs predicted mortality, we created a prognostic index giving a score of 1 for: respiratory rates ≥30 cycles/minute; pulse rates ≥100 beats/minute; mean arterial pressures ≥110/<70 mmHg; temperatures ≥38.6/<35.6°C; and presence of altered mental state defined as Glasgow coma score ≤14; 0 for all other values. The proposed index (maximum score = 5) predicted mortality comparably to MEWS. Patients scoring ≥3 on the index were 3.4-fold (95% confidence interval (CI) 1.6 to 7.3, *P* = 0.001) and 2.3-fold (95% CI 1.1 to 4.7, *P* = 0.031) as likely to die in hospital as those scoring 0 to 2 in the development and validation cohorts respectively; those scoring ≥5 on MEWS were 2.5-fold (95% CI 1.2 to 5.3, *P* = 0.017) and 1.8-fold (95% CI 0.74 to 4.2, *P* = 0.204) as likely to die as those scoring 0 to 4 in the development and validation cohorts respectively.

**Conclusion:**

Among patients with sepsis, a prognostic index incorporating admission vital signs data with reduced segmentation in the values of included variables adequately predicted mortality. Such an index may be more easily implemented when triaging acutely-ill patients. Future studies using a similar approach may develop indexes that can be used to monitor treatment among acutely-ill patients, especially in resource-limited settings.

## Introduction

In sub-Saharan Africa (SSA), sepsis is common and is associated with high mortality [1]. Also, available treatment interventions and tools to guide their administration are few. Although studies are increasingly being performed in the region to test various interventions, the design of such studies is affected by lack of monitoring tools [2].

In resource-rich environments, vital parameters such as the central venous pressure (CVP), mean arterial pressure (MAP), and central venous oxygen saturation (ScvO2) commonly guide treatment of patients with sepsis [3]. Initial values of these variables and subsequent changes thereof are considered when deciding whether or not to give, escalate, or de-escalate interventions such as intravenous fluids, diuretics, supplemental oxygen, blood transfusion, and vasopressors or, if needed, vasodilators [4]. As these variables are commonly measured using invasive techniques, and also their prognostic properties are not fully understood, their use in guiding treatment continues to be challenged [5,6]. Additionally, it is difficult to use such variables to monitor treatment in resource-limited settings, where monitoring facilities are scarce.

Better tools to monitor treatment of patients with sepsis are therefore required in all settings. However, this need is more acute in SSA, where any mildly sophisticated monitoring tools are rarely available, and critically-ill patients are treated from general wards [7,8]. In this environment, routine vital signs are among the feasible options to monitor treatment. Routine vital signs are particularly appealing for this purpose; they represent a treatment-modifiable signal that can be obtained using cheaper and non-invasive techniques. However, their prognostic utility has not been adequately studied.

A number of existing prognostic indexes incorporate vital signs data [9-11]. As these indexes predict post-hospitalization mortality [10-12], they can be used to triage acutely-ill patients, which is how they are predominantly used in resource-rich settings [13]. Such indexes could also be used to monitor treatment, especially in resource-limited settings like SSA, if their prognostic properties were adequate, which is not the case [14]. Although they predict mortality [10,15], their accuracy and efficiency remain inadequate [16], and indexes like the Modified Early Warning Score (MEWS), where the values of included variables are heavily segmented [17,18], can be difficult to implement in routine clinical settings. It is not clear if the segmentation in the MEWS can be reduced while preserving prognostic properties.

Recent studies in SSA have focused on validating less-segmented prognostic indexes. In a recent study in Malawi, two alternative indexes; one incorporating hypotension, oxygen saturation, temperature, electrocardiographic abnormality, and loss of independence (HOTEL); and another, incorporating tachypnea, oxygen saturation, temperature, alertness, and loss of independence (TOTAL), performed better than MEWS in predicting mortality [14]. However, these indexes also contain variables that are not always available, for example the peripheral oxygen saturation, and variables whose prognostic abilities are not adequately understood, for example loss of independence, and include some variables in ways that may not be efficient, for example including both tachypnea and oxygen saturation.

Among patients hospitalized with sepsis, we aimed to describe how admission vital signs data predict mortality. We hoped to use this information to create a prognostic index incorporating vital signs data with less segmentation in the values of included variables. Improved prognostic indexes that use vital signs data may be used to monitor treatment of patients with sepsis.

## Methods

We analyzed data of adults hospitalized with sepsis, who were enrolled in two cohort studies at the Mbarara Regional Referral Hospital in Mbarara, Uganda. Using admission vital signs data in one cohort (the development cohort), we constructed a prognostic index to predict mortality. To determine how the index would perform in a different patient group, we assessed its performance in another cohort (the validation cohort). The setting and study populations of the two cohorts have been described elsewhere [8,19]. Briefly, the first study occurred in April to June 2011 and prospectively enrolled adults hospitalized for any medical illness [19]. From the patients enrolled in this study (n = 318), we selected those meeting criteria for sepsis at admission (n = 167), who comprised the development cohort. All patients enrolled in this cohort provided written informed consent to participate. The second study occurred in February to July 2009 and had prospectively enrolled patients meeting criteria for sepsis (n = 150) [8], who comprised the validation cohort. These patients also had provided written informed consent to participate. Both studies were approved by the Institutional Review Committee (IRC) at Mbarara University of Science and Technology.

### Measurements

Study definitions and procedures for measuring vital signs were similar across the two cohorts. We defined sepsis and severe sepsis using clinical definitions, since definitions based on complex laboratory testing would be difficult to implement in this setting. Accordingly, sepsis was defined as the presence of suspected infection, plus two or more of the systemic inflammatory response syndrome (SIRS) criteria (pulse ≥90 beats/minute; respiratory rate ≥20 cycles/minute; a temperature ≥38°C or ≤36°C; and white blood cell count ≥12,000 cells/cc, or <4,000 cells/cc, or >10% band forms). Severe sepsis was defined as the presence of sepsis plus at least one organ dysfunction (Glasgow coma score (GCS) <15, systolic blood pressure (SBP) <90 mmHg, or platelet counts <100,000 cells/cc) [20]. Blood pressures were measured using standard techniques and a manual cuff; all temperatures were axillary, and pulse and respiratory rates were obtained by manual counting. We calculated the MAP as a weighted average of the SBP and the diastolic BP (DBP) (SBP plus twice the DBP divided by 3) [21] and assessed mental state using the GCS. For all variables, the admission value was the first measurement obtained from the patient on their day of admission.

### Determination of in-hospital mortality

In both cohorts, patients were followed in hospital until death or discharge, and there were no losses to follow-up.

### Analysis

#### Descriptive summaries

We first summarized patient characteristics in each cohort using medians and inter-quartile ranges (IQR) for vital signs, as well as appropriate summaries for age, sex, severity of sepsis, mental state, and suspected focus of infection. We proceeded to assess relationships between vital signs and mortality and to develop and validate the proposed prognostic index as described below.

#### Development of prediction rules and the prognostic index

In the development cohort, we used restricted cubic splines of admission vital signs data to predict the probability of in-hospital death; restricted cubic splines are a flexible way of assessing relationships [22-24]. Based on the shapes of smoothed curves of vital signs data against probability of in-hospital death, we created prognostic and reference categories in the values of each vital sign. We defined reference categories as those with low mortality; that is below or about the sample average (23%). Prognostic categories were those where mortality was above this average. We then calculated average mortality in each category. Using this information, we determined cutoffs in the values of each vital sign below or above which a score of 1 would be given in a simple scoring system to create a prognostic index. For mental state data, we classified patients by GCS level as: ≤12, 13 to 14, and 15. Guided by the distribution of mortality in these categories, we also created a binary cutoff for GCS. We assessed the performance of the proposed index in the development cohort by calculating proportions dying at different scores, as well as unadjusted odds ratios (OR) and 95% confidence intervals (CI) comparing mortality in subjects with scores ≥3 to mortality in those with scores of 0 to 2.

#### Validation of prediction rules and the proposed index

To better understand whether the prediction patterns observed in the development cohort could be replicated in a different patient group, we created similar reference and prognostic categories in the values of each vital sign in the validation cohort. Further, we assessed the performance of the proposed index in the validation cohort in the same way that we assessed its performance in the development cohort (that is, by calculating proportions dying at different scores, as well as unadjusted ORs and 95% CI comparing mortality in subjects with scores of ≥3 to mortality in those with scores of 0 to 2). In both cohorts, we also compared the performance of the proposed index to that of the MEWS.

### Statistical issues

In the development cohort, we imputed 11 to 34 missing values on respiratory rate, white cell counts, and GCS using multiple imputation (MI) techniques and the other variables with complete data [25,26]. We then used 20 extracted and averaged post-MI datasets to perform all analyses of the development cohort. The medians and IQRs for the respiratory rate, white cell counts, and GCS in this cohort, as well as the restricted cubic spline models, were therefore obtained from post-MI datasets. There were no missing data in the validation cohort.

## Results

### Subject characteristics

#### Development cohort

We included in the development cohort 167 patients with sepsis (61% had severe sepsis) hospitalized in April to June 2011. Median age was 38 (IQR 28 to 55). Fifty-three percent were male, and the largest proportion (60%) had suspected chest infection. In this cohort, in-hospital mortality was 23% overall, and 29% in those with severe sepsis. Overall median length of stay in hospital was 6 days (IQR 6 to 10).

#### Validation cohort

This cohort comprised 150 patients with sepsis (65% had severe sepsis) hospitalized in February to July 2009. Median age was 31 (IQR 25 to 41). Sixty-three percent were male, and the largest proportion also had suspected chest infection (64%) (Table 1). In-hospital mortality was 30% overall, and 41% in those with severe sepsis. Overall length of stay in hospital was 4 days (IQR 1 to 7).

**Table 1:** **Characteristics at admission of 317 patients with sepsis hospitalized in 2009 and 2011 in Uganda**

**Variable**	**Development cohort (n = 167)**	**Validation cohort (n = 150)**
**Numeric variables, median (IQR)**		
Age	38 (28 to 55)	31 (25 to 41)
Pulse (beats/minute)	108 (90 to120)	110 (104 to 120)
Temperature (°C)	37 (36.5 to 38.3)	38.5 (38 to 39)
Respiratory rate (cycles/minute)	28 (22 to 32)	30 (24 to 36)
Systolic blood pressure (mmHg)	100 (90 to 120)	110 (85 to 110)
Diastolic blood pressure (mmHg)	60 (50 to 70)	60 (50 to 70)
Mean arterial pressure (mmHg)	73 (63 to 87)	73 (63 to 88)
White cell counts (× 10^3^ cells/cc)	4.7 (2.7 to 6.2)	5.7 (3.2 to 9.2)
Platelets (× 10^3^ cells/cc)	160 (108 to 229)	164 (88 to 252)
**Binary variables, n (%)**		
Sex male	88 (53%)	94 (63%)
Altered mental state	35 (21%)	35 (23%)
Severe sepsis	101 (61%)	97 (65%)
Suspected focus of infection		
Chest	100 (60%)	96 (64%)
Central nervous system	45 (27%)	18 (12%)
Gastro-intestinal	2 (1.2%)	17 (11%)
Other focus	20 (12%)	19 (13%)

The table shows the characteristics of patients admitted with sepsis at Mbarara Regional Referral Hospital in Uganda. One hundred and sixty-seven patients hospitalized in April to June 2011 were included in the development cohort, and 150 patients with sepsis hospitalized in February to July 2009 were included in the validation cohort. IQR, inter-quartile range.

#### Relationships of admission vital signs data with mortality

**Figure 1:**
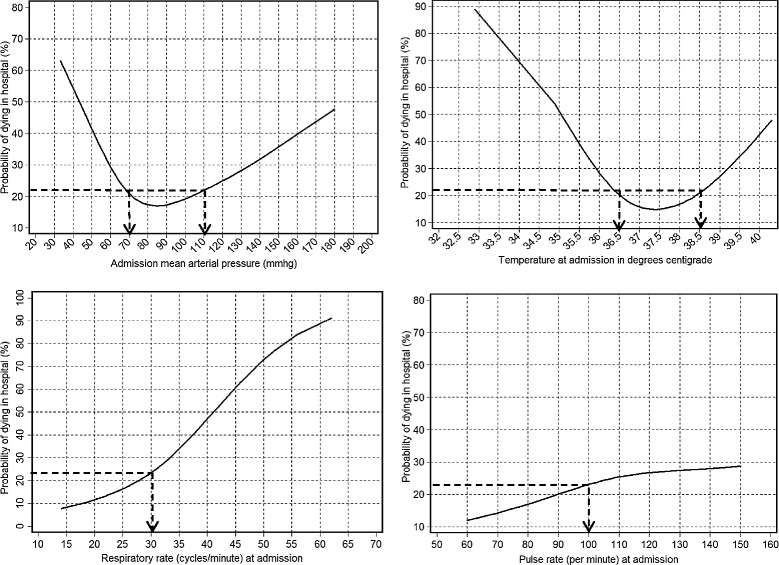
**Probability of in-hospital death by admission mean arterial pressure, temperature, and respiratory and pulse rates.** The figure shows smoothed predictions for the probability of in-hospital death according to the admission mean arterial pressure (MAP), temperature, and respiratory and pulse rates among 167 patients hospitalized with sepsis in April to June 2011 in Uganda. Admission MAP and temperature predicted mortality with approximately U-shaped relationships; mortality increased as either variable increased or decreased starting from a reference range (70 to 109 mmHg for MAP and 36.6 to 38.5°C for temperature). Admission respiratory and pulse rates predicted mortality in roughly linear relationships; at respiratory rates ≥30 cycles/minute mortality appeared to increase approximately linearly as respiratory rate increased. For the pulse rate, mortality did not go above average until a pulse rate ≥100 beats/minute and tapered off close to the average mortality for this population (about 23%) as the pulse rate increased. Using these patterns, we determined cutoffs (shown by arrows on the figure) below or above which we gave a score of 1 in the proposed prognostic index.

Admission MAP and temperature predicted mortality with approximately U-shaped relationships; pulse and respiratory rates predicted mortality in roughly linear relationships (Figure 1). Altered mental state also predicted mortality; mortality was 40%, 33%, and 20% in patients with GCS ≤12, 13 to 14, and 15, respectively. Consequently, we determined that the prognostic values for the GCS variable would be ≤14; patients with GCS ≤14 were 2.4-fold (95% CI 1.1 to 5.4, *P* = 0.033) as likely to die as those with GCS of 15.

#### Prediction rules for constructing the proposed prognostic index

Using the shapes of the graphs (Figure 1) and how altered mental state predicted mortality, we determined the following rules: first, that a scoring system giving a score of 1 for high respiratory rate (≥30 cycles/minute), high pulse rate (≥100 beats/minute), high or low MAP (≥110 or <70 mmHg), high or low temperature (≥38.6°C and <35.6), and altered mental state (GCS ≤14), and 0 for all other values (Table 2), would adequately predict mortality; second, that in such an index, low values of respiratory and pulse rates may also be given a score of 0.

**Table 2:** **Abnormalities in vital signs to receive a score of 1 in a proposed prognostic index**

**Vital sign**	**Abnormality**	**Cutoff** ^*^
Respiratory rate (cycles/minute)	High	≥30
Mean arterial pressure	High or low	≥110 or <70
Temperature (°C)	High or low	≥38.6 or <35.6
Pulse rate (beats/minute)	High	≥100
Altered mental state/GCS	Low GCS	GCS ≤14

^*^The proposed index gives a score of 1 for the indicated ranges and a score of 0 for all other values. The cutoffs are based on how each of the vital signs predicted mortality in the development cohort. GCS, Glasgow coma score.

#### Validation of the proposed prediction rules

In assessing whether the proposed prediction rules were reliable, patterns of mortality in the prognostic categories of different vital signs were roughly similar across the two cohorts (Table 3).

**Table 3:** **In-hospital mortality in prognostic categories of different vital signs**

	**Development cohort (N = 167)**		**Validation cohort (N = 150)**	
**Variable**	**n** ^*^	**Mortality (%)**	**n**	**Mortality (%)**
Temperature (°C)				
≤36.5	51	28%	17	53%
36.6 to 38.5 (reference)	84	19%	67	30%
≥38.6	32	28%	66	24%
Mean arterial pressure (mmHg)				
≤69	56	30%	52	39%
70 to 109 (reference)	86	19%	95	25%
≥110	13	23%	3	33%
Respiratory rate (cycles/minute)				
<30 (reference)	102	15%	65	28%
≥30	65	37%	85	32%
Pulse rate (beats/minute)				
<100 (reference)	61	20%	16	25%
≥100	106	26%	134	31%
Glasgow coma score				
15 (reference)	133	20%	115	23%
14 to 13	24	33%	13	39%
≤12	10	40%	14	64%

^*^Number of patients in category. The table shows mortality in prognostic categories of different vital signs among patients with sepsis hospitalized in Uganda. The individual categories were created according to how vital signs predicted mortality in the development cohort.

#### Prediction of mortality by the proposed index

The prognostic index (total possible score = 5) developed according to the above rules (Table 2) adequately predicted mortality in the development and validation cohorts; mortality increased with increasing scores of the proposed index. For example, mortality was 11% at a score of 0, increasing to 25% at a score of 2 and to 36% at score of 3 in the development cohort; mortality was 27% and 16% at scores of 1 and 2 respectively, increasing to 34% at a score of 3 in the validation cohort (Table 4).

**Table 4:** **Mortality at different scores of the proposed prognostic index**

	**Development cohort**		**Validation cohort**	
**Score**	**Number of patients**	**Died (%)**	**Number of patients**	**Died (%)**
**0**	28	11%	3	33%
**1**	32	6.3%	26	27%
**2**	57	25%	38	16%
**3**	36	36%	59	34%
**4**	12	42%	18	33%
**5**	2	100%	6	83%

The table shows the number of patients and proportions that died in hospital at increasing scores of the proposed prognostic index by cohort among patients with sepsis hospitalized in Uganda.

#### Performance of the proposed index and MEWS in the development cohort

The proposed prognostic index performed well and comparably to MEWS in the development cohort; patients scoring ≥3 on the proposed index were 3.4-fold (95% CI 1.6 to 7.3, *P* = 0.001) as likely to die in hospital as those scoring 0 to 2; those scoring ≥5 on MEWS were 2.5-fold (95% CI 1.2 to 5.3, *P* = 0.017) as likely to die as those scoring 0 to 4 (Table 5).

**Table 5:** **Prediction of mortality by the Modified Early Warning Score (MEWS) and the proposed index**

	**Development cohort (N = 167)**		**Validation cohort (N = 150)**	
**Score**	**OR (95% CI)**	***P***	**OR (95% CI)**	***P***
**Proposed index**				
0 to 2	Ref	-	Ref	-
≥3	3.4 (1.6 to 7.3)	0.001	2.3 (1.1 to 4.7)	0.031
**MEWS**				
0 to 4	Ref	-	Ref	-
≥5	2.5 (1.2 to 5.3)	0.017	1.8 (0.74 to 4.2)	0.204

The table shows unadjusted odds ratios and 95% confidence intervals of in-hospital death at scores ≥5 for MEWS and at scores ≥3 for the proposed index among patients with sepsis hospitalized in Uganda. OR, odds ratio; CI, confidence interval; MEWS, Modified Early Warning Score.

### Performance of the proposed index and MEWS in the validation cohort

The proposed index also performed well and comparably to MEWS in the validation cohort; patients scoring ≥3 on the proposed index were 2.3-fold (95% CI 1.1 to 4.7, *P* = 0.031) as likely to die as those scoring 0 to 2; those scoring ≥5 on MEWS were 1.8-fold (95% CI 0.74 to 4.2, *P* = 0.204) as likely to die as those scoring 0 to 4 (Table 5).

## Discussion

In SSA, routine vital signs are among the feasible options to monitor treatment of critically ill patients. Among patients hospitalized with sepsis in Uganda, we used restricted cubic splines and plotted smoothed curves of admission vital signs data against the probability of in-hospital death. Using this information, we determined rules to create a composite prognostic index incorporating vital signs data. Our findings suggest that prognostic indexes may include vital signs data with reduced segmentation in the values of included variables, and that low values of respiratory and pulse rates may not be scored. A proposed prognostic index based on these rules and following a similar principle as MEWS [18] but with less segmentation adequately predicted mortality and compared favorably to MEWS.

Prognostic indexes using vital signs data usually contain substantial segmentation in the values of incorporated variables. For example, MEWS gives: 3 points for each of SBP <70 mmHg, pulse rate ≥130 beats/minute, respiratory rate ≥30 cycles/minute, and GCS score ≤8; 2 points for each of SBP 70 to 80 or ≥200 mmHg, pulse 111 to 129 beats/minute, respiratory rate <9 or 21 to 29 cycles/minute, temperature <35 or ≥38.5°C, and GCS 9 to 13; 1 point each for SBP 80 to 100 mmHg, pulse rate 40 to 50 or 101 to 110 beats/minute, respiratory rate 16 to 20 cycles/minute, and GCS score of 14; and 0 for all other values [18]. A recent modification suggested for use in SSA includes more variables (seven in total) and more segmentation in some variables [17]. The high degree of segmentation may affect the efficiency and reliability of these indexes [27] and, in routine clinical settings, their applicability.

Our data suggest that less segmentation may be achieved in at least four vital signs. In blood pressure (BP) and temperature, average mortality on either side of the reference categories did not differ substantially. Although extreme values may be associated with higher mortality than intermediate values, the data in the extreme ranges are also likely to be thin most of the time. For respiratory rate and pulse, mortality did not go above average until respiratory rate ≥30 cycles/minute and pulse rate ≥100 beats/minute respectively. In addition, low respiratory rates were rare (in both cohorts, no patient had respiratory rate <12 cycles/minute). We interpret these patterns to mean that in composite prognostic indexes using vital signs data, low and high values of BP and temperature may be scored similarly, and that for pulse and respiratory rate, scoring only the high values may be adequate.

Many prognostic scoring systems have been developed for monitoring critically-ill patients. These include single-point-in-time-instruments like the Acute Physiology and Chronic Evaluation (APACHE) [28] and progressive instruments like the Sequential Organ Failure Assessment (SOFA) [29]. However, these tools remain less applicable in acute care settings, where patients spend limited time. They are even less applicable in resource-limited settings, where support systems like laboratories are weak. Scoring systems like MEWS that use simple, yet treatment-modifiable variables, are therefore a welcome addition. However, there are not enough data supporting their use in both triage and monitoring. Our results represent one approach to creating newer/simpler triage/monitoring systems.

Monitoring vital signs in acutely ill patients can also aid integrated management of acute and chronic illnesses. For example, in our data, patients with high BP at admission had higher mortality than those with normal BP. As hypertension in sepsis is not usually associated with high mortality [30], this finding may suggest treatment opportunities that could be addressed by better vital signs monitoring, for example being careful with fluid administration in patients with pre-existing hypertension, while still giving appropriate fluid resuscitation to those who may have elevated BP at admission but without pre-existing hypertension.

Our findings suggest that studies using vital signs data to monitor outcomes should consider modeling these data more flexibly, yet efficiently. Previous studies have reported relationships between BP and mortality in binary fashion, for example mortality at MAP <60 mmHg versus ≥60 mmHg, or in incremental categories, for example mortality per 10 unit increase in BP starting from a reference category [8,31]. We suggest that future studies consider the approximately U-shaped patterns in the relationships of mortality with both BP and temperature and the roughly linear patterns for respiratory and pulse rates. These patterns should be considered when measuring vital signs at one point in time, for example at admission, or progressively, for example at post-admission intervals [32].

Our findings have some limitations. As vital signs were infrequently measured, we were not able to use multiple measurements for each vital sign, in addition to imputing some missing values in the development cohort, which may affect the precision of our measurements. Restricted cubic splines are a flexible way of assessing relationships. However, the resulting curves are smoothed, and, especially at the extremes, where data are usually thin, the predictions can be unreliable. As the sample sizes were small, the OR estimates, especially in the validation cohort, had wide 95% CIs and, for the estimates assessing the performance of MEWS, a *P* value not reaching statistical significance. Also, binary modeling, which gives an equal score at all GCS levels, may lead to loss of information. Future studies should examine more efficient, yet still simple, ways of modeling not only GCS, but also other vital signs. Despite these limitations, our findings have potential to guide the development of prognostic indexes incorporating vital signs data that can be used to monitor treatment. We included in the proposed index only treatment-modifiable variables, and, despite the small sample sizes, prediction patterns in the development cohort were replicated in the validation cohort.

## Conclusions

Among patients hospitalized with sepsis in sub-Saharan Africa, a prognostic index incorporating vital signs data with less segmentation in the values of included variables and giving no score for low values of respiratory and pulse rates adequately predicted mortality. Such an index may be easier to implement than existing related indexes when triaging acutely-ill patients. In addition, future studies using a similar approach on post-admission vital signs may develop prognostic indexes that can be used to monitor treatment among acutely-ill patients, especially in resource-limited settings.

## Key messages

- Existing prognostic indexes that use vital signs information incorporate this information in ways that are not always efficient
- We used restricted cubic splines to assess how admission vital signs data predict mortality in patients with sepsis
- Guided by these predictions, we incorporated treatment-modifiable routine vital signs into a prognostic index with reduced segmentation in the values of incorporated variables
- The resulting prognostic index adequately predicted mortality in two cohorts of patients with sepsis
- Future studies using a similar approach on post-admission vital signs data could develop indexes that can be used to monitor treatment, especially in resource-limited settings

## Abbreviations

BP: blood pressure
CI: confidence interval
CVP: central venous pressure
DBP: diastolic blood pressure
GCS: Glasgow coma score
HOTEL: hypotension, oxygen saturation, temperature, electrocardiographic abnormality, and loss of independence
IQR: inter-quartile range
IRC: Institutional Review Committee
MAP: mean arterial pressure
MEWS: Modified Early Warning Score
MI: multiple imputations
OR: odds ratio
SBP: systolic blood pressure
SIRS: systemic inflammatory response syndrome
SSA: sub-Saharan Africa
ScvO2: central venous oxygen saturation
TOTAL: tachypnea, oxygen saturation, temperature, alertness, and loss of independence
